# Prevalence of Moderate to Severe Periodontitis in an 18–19th Century Sample—St. Bride’s Lower Churchyard (London, UK)

**DOI:** 10.3390/dj10040056

**Published:** 2022-04-01

**Authors:** Ruqayah Al-Mutairi, Helen Liversidge, David Geoffrey Gillam

**Affiliations:** 1 Al Jahra Speciality Dental Center, Ministry of Health, Al Qasser, Block 2, Street 619, Al Jahra 00004, Kuwait; rhmutairi@hotmail.com; 2 Oral Bioengineering, Institute of Dentistry, Barts and the London School of Medicine and Dentistry QMUL, London E1 2AD, UK; h.m.liversidge@qmul.ac.uk

**Keywords:** prevalence, periodontitis, case definition, post-medieval population, Romano-British population, human skulls, dental pathologies

## Abstract

The aim of the study was to determine the prevalence of moderate to severe periodontitis in 18–19th century skulls in the St Bride’s Lower Churchyard in London, UK. Materials and methods: A total of 105 adult skulls (66 M: F 39) from the Museum of London collection were examined for evidence of dental disease. The primary method was to measure the presence of moderate to severe periodontitis. Other dental pathologies were recorded such as tooth wear, calculus, and caries. Results: Overall, the prevalence of moderate to severe periodontitis in the sample was 21–24%. Males were observed to be more susceptible to periodontal disease than females. The severity of bone loss in the skull collection also increased with age. There was no significant difference in the amount of calculus deposition when comparing either age or sex. A total of 14% of the individuals in the sample showed signs of smoking. Conclusion: The results of the study indicated that the prevalence of moderate to severe periodontitis in an 18–19th century skull sample was 21–24%, which was higher than in previous studies. This may be due to the lack of basic personal mouth care and professional dental treatment as well as known risk factors such as smoking, stress, low socioeconomic status, and malnutrition.

## 1. Introduction

Periodontitis is a global disease affecting over 50% of the world’s population and is currently the sixth most prevalent human disease [1]. Periodontitis may impact on the population depending on its severity; for example, 11.2% suffer from the severe form of periodontitis, whereas both mild and moderate periodontitis affects most adults in the population [2]. The main aetiological factor for periodontal disease is a dental biofilm, although the extent and severity of the disease is determined by the host response in association with risk factors such as smoking, obesity, and diabetes. According to Raitapuro-Murray et al. [3] the study of the epidemiology of periodontal disease together with other dental disease in ancient populations is useful for determining (1) the prevalence of the disease, (2) the extent and severity of the disease, (3) the natural history of the condition, and (4) the aetiology of the disease.

Several methods have been used to assess the prevalence of periodontal disease in previous (past) populations such as dried skulls/radiographical examination [3,4,5]. Brothwell [6] suggested using categories of none, mild, moderate, and severe periodontitis depending on the degree of root exposure. These techniques, however, relied on a subjective interpretation and made inter-observer and inter-study comparisons difficult [7]. Davies and Picton [8] reported that the cementum-enamel junction (CEJ) should be used as a fixed level of reference to determine the amount of bone loss on both the buccal/labial and mesial aspects of the teeth. This method was considered more objective and facilitated inter-study comparisons, although the linear measurement of the distance between CEJ and alveolar bone (AC) has been criticised since it ignored the continuous eruption of the teeth and consequently overestimated the prevalence of periodontitis [3].

Several studies have attempted to estimate the level of periodontal disease in a UK sample population, for example, Raitapuro-Murray et al. [3] reported that the prevalence of moderate to severe periodontitis in a Romano-British population 200–400 AD was low (5.6%), whereas Goncalves et al. [9] reported that the severity of periodontal disease had declined in the United Kingdom from the 3rd–5th (III-V) century to the 18th (XVIII) century.

## 2. Aim of the Study

The aim of the study was to determine the prevalence of moderate to severe periodontitis in the skeletal remains of 105 adults from the 18–19th century (1770–1849) buried in the St Bride’s Lower Churchyard in London, UK. A secondary aim of the study was to compare the results of the studied population with those recorded from human skulls in a Romano-British population (200–400 A.D.) [3].

## 3. Materials and Methods

### 3.1. The Sample

One hundred and five skulls were examined from a total of 544 human skeletal remains of a post-medieval population of St Bride’s Lower Churchyard cemetery with a total of 1721 teeth. The cemetery dates from the late 18th and 19th century (1770–1849). St Bride’s Lower Churchyard cemetery was located at 72–82 Farringdon Street and included skeletal remains due to overcrowding from the actual churchyard linked to St Bride’s Church in Fleet Street (London, UK). The archaeological excavation of the burial ground was performed by the Museum of London Archaeological Services in 1990 and the skeletal collection subsequently retained and curated as part of the archaeological archive at the Centre for Human Bioarchaeology (CHB). Furthermore, all the skeletal remains from the site were analysed and recorded on the Oracle osteological database following the methods which are downloadable from the CHB website [10,11]. Sex was determined by the museum staff using general cranial and pelvic morphology; all information pertaining to this collection was obtained through an online database provided by Museum of London. Most of the skeletal remains were buried in wooden coffins and around 95% of these coffins were well preserved. The cemetery contained individuals who were mainly from a lower socioeconomic class from either (1) the Bridewell workhouse or (2) the Fleet Street prison (Miles and Conheeney [12]).

### 3.2. Inclusion/Exclusion Criteria

Skulls were excluded if (1) the individual was under 18 years of age, (2) if the individual was unable to be assigned to a group or if the age and/or sex could not be determined, (3) where there was extensive post-mortem damage or fracture of the skeletal remains, and (4) if the skull was edentulous.

### 3.3. Data Collection

Each skull was carefully examined by one of the authors (RA) prior to data collection for any sign of damage, post-mortem trauma, or pathology (Table 1).

To clarify whether teeth were missing or simply displaced prior to death (ante-mortem) or after death (post-mortem) the following guidelines were adhered to: (1) teeth were recorded ‘lost after death’ (post-mortem) if there was an empty alveolus with no sign of remodelling and (2) teeth lost post-mortem were carefully placed back into the socket closest to their original position as possible. Teeth were recorded as ‘before death’ (ante-mortem) if on examination there were traces of healing and remodelling of the edge of the tooth socket and infilling of the affected socket with new bone [13].

Bone loss was recorded following the methodology reported by Raitapuro-Murray et al. [3]. A Hu-Friedy UNC 15 periodontal probe with a 1 mm gradation was used in the measurement of the bone and recorded in millimetres (Raitapuro-Murray et al. [3]). The estimation of horizontal bone loss was performed by measuring the distance between the alveolar bone crest (AC) and CEJ in six sites of all existing teeth which did not have post-mortem bone defects as described by Raitapuro-Murray et al. [3] and Goncalves et al. [9]. An estimation of the vertical bone loss was performed by measuring the distance between the CEJ and the base of the defect when an infrabony defect was present in the alveolar bone. The measurements recorded represented an estimation of bone loss and, therefore, cannot be viewed as an accurate representation of periodontal disease progression. The bone loss pattern was recorded both buccally and lingually for each tooth as horizontal and/or vertical. Third molars were excluded for the purposes of evaluation.

Dental pathologies and anomalies that may have affected the individual prior to death (caries, calculus, tooth wear) were also recorded as either present or absent (Table 2).

Occlusal tooth wear measurements were recorded for each tooth using the Raitapuro-Murray et al. [3] index. Tooth surface loss was scored as following: 0 = none, 1 = visible, 2 = flattened cusp, 3 = extensive with dentine exposure

## 4. Reproducibility

To assess intra-examiner calibration, calibration blocks with pre-determined probing depths were used prior to the commencement of the study. During the study five skulls (4.7%) were also randomly chosen for rerecording to assess intra-examiner reproducibility (RA).

## 5. Data Analysis/Case Definition

Both sex and age of the skeletal remains were pre-determined by the Centre of Human Bioarcheology staff in the Museum of London. The samples were divided into four age groups namely (1) 18–25 years, (2) 26–35 years, (3) 36–45 years, and (4) 46 years and older.

## 6. Periodontal Disease

Calculation of the average value of CEJ-AC per tooth was based on measurements from the previous six sites around a tooth to determine the mean value per skull and age group using the methodology described by Goncalves et al. [9]. Skulls with horizontal bone loss ≥ 2 mm above the mean (per tooth) for the appropriate age were considered as ‘periodontitis’ [3,9,14]. The chosen threshold of 2 mm was to allow for any possible variance within the expected rate of continuous eruption of teeth [3].

### 6.1. Cases of Moderate to Severe Periodontitis

#### 6.1.1. Case Definition I

Teeth were identified as being periodontally involved (periodontitis) based on the total measured bone loss in the vertical dimension. For example, to qualify as a tooth being periodontally involved, the bone loss should be ≥5 mm above the age average. Criteria for defining periodontitis in the different age groups was defined by Raitapuro-Murray et al. [3] to allow for comparison of the extent of disease which may be clinically diagnosed as moderate or severe periodontitis. Determination of the severity of periodontitis (moderate/severe) was based on the 1999 American Academy of Periodontology (AAP) classification of periodontal disease [15].

Age groups were modified based on the available date for inclusion in the present study:

18–25 years old   2 or more teeth affected

26–35 years old   3 quadrants affected

36 years old and older  4 quadrants affected

#### 6.1.2. Case Definition II

Three or more affected sites with at least 5 mm bone loss, independent of the number of quadrants affected [3].

## 7. Results

The skeletal remains from the original collection (*n* = 516) were excluded from inclusion for the following reasons: (1) 228 missing skulls, (2) 43 adults were unclassified or under 18 years of age, (3) 84 adults were either edentulous or had post-mortem damage, and (4) 56 adult skulls were unexamined due to time constraints. The sample in the present study, therefore, consisted of the skeletal remains of 105 individuals (66 M:39 F) with a total of 1721 permanent teeth. Most of the skulls were in good condition although the skulls were stained with a dark brown colour due to decomposition and exposure to acid and minerals. Evidence of post-mortem damage was present particularly on the buccal surfaces of the jaws. There was no visible evidence of dental treatment provided for this population apart from the extraction of teeth, although one individual received treatment for a gold restoration on the occlusal surface of one molar tooth.

### Calibration/Reproducibility

An intra-operator reproducibility study was completed by RA for all recording indices. The agreement between the two measurements of horizontal bone loss showed a high correlation (buccal bone loss 89.5%, lingual/palatal bone loss 93.3%; % mean 91% respectively).

## 8. Tooth Status

The status of 3149 teeth was recorded and 1947 teeth were suitable for further evaluation. Following the exclusion of third molars and teeth unable to be relocated into the tooth socket, 1721 teeth were included in the final analysis. The number of teeth lost during the lifetime of individuals in the sample is shown in Table 3. An average loss of 8.8 teeth occurred during the lifetime of individuals over 46 years of age. Table 4 describes the number of individuals with post-mortem tooth loss in each age group.

Approximately 81% of the teeth were lost post-mortem from which 69% were successfully returned to their original position in the respective jaws by RA.

## 9. Alveolar Bone Loss

**Figure 1:**
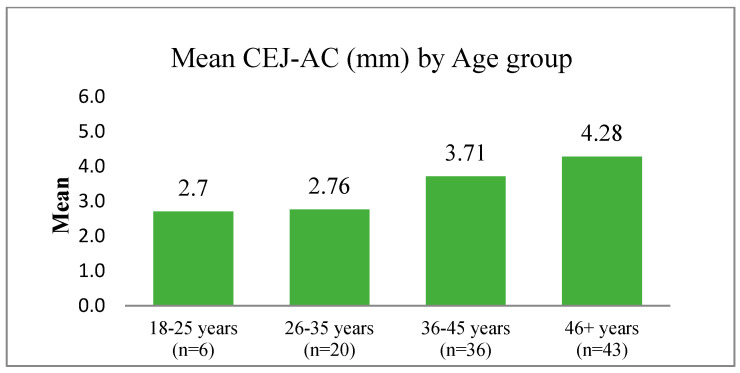
Mean CEJ–AC (mm) (the mean CEJ-AC distance by age group for all teeth (*n* *=* 1721)).

**Figure 2:**
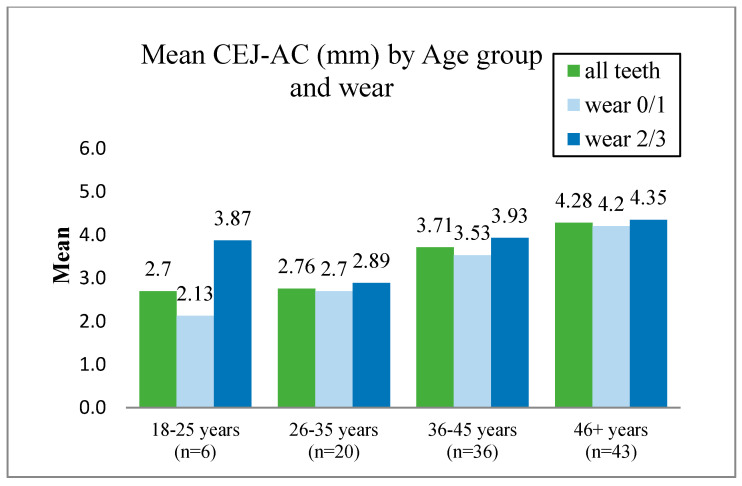
Mean CEJ–AC (mm) by tooth wear (mean CEJ-AC distance by age group for all teeth, teeth with wear 0/1, and teeth with wear 2/3).

The methodology reported by Goncalves et al. [9] was used to measure the alveolar bone loss by recording the mean CEJ-AC from six points (buccal—mesial, mid, distal—and palatal and lingual—mesial, mid, distal—surface(s) for each tooth was calculated) (Figure 1). The tooth wear level was classified into two groups: 0/1 and 2/3. Figure 2 displays the bone loss distribution data according to this classification.

A two-way ANOVA model was used to compare the CEJ-AC means by age and wear. Significant differences were obtained between the age groups (*p* < 0.001, F-test), between the wear level groups (*p* < 0.001, F-test) and by comparing age with wear (*p* < 0.001, F-test).

For age groups 18–25 and 36–45 years, differences reached statistical significance (*p* < 0.001, B., *p* = 0.002, B.). No differences were observed for age groups 26–35 years (*p* = 0.310, B.) and for 46+ years (*p* = 0.243). The exception was in the age group 18–25 years old, where teeth with a higher level of wear (dark blue columns) were present with similar mean levels of CEJ-AC distance when compared to teeth with a lower wear grade (Figure 2, light blue columns).

## 10. Horizontal Bone Loss

**Figure 3:**
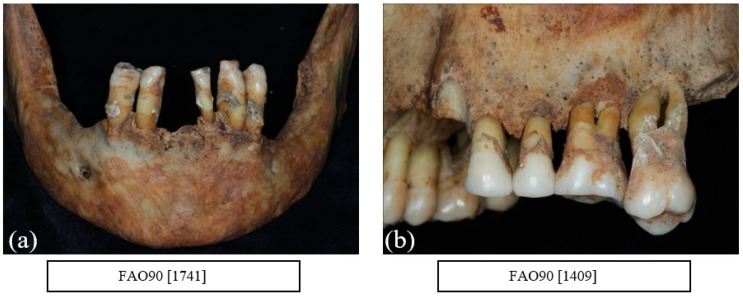
Illustration of generalised moderate to severe horizontal bone loss affecting both the anterior teeth in the mandible (lower jaw) ((**a**) FAO90[1741]) and the posterior teeth in the maxilla (upper jaw) ((**b**) FAO90[1409]) (courtesy of the Museum of London).

A total of 31.1% of the sample was affected with horizontal bone loss. Table 5 summarises the horizontal bone loss by age and sex. More males (*n* = 27) were numerically affected with horizontal bone loss than females (*n* = 12) with 40.9% and 30.8% individual bone loss respectively (Figure 3a,b).

According to Case definition I, 21% of adults were affected with moderate to severe periodontitis (*n* = 22). Older adults showed a higher prevalence compared to the rest of the age groups (*p* < 0.001). No differences were observed between males and females (22.7%, 17.9%) (*p* = 0.899) (Table 6).

According to case definition II, 24.8% were affected with moderate to severe periodontitis (*n* = 26). There were no statistically significant differences between the age groups. However, there was a statistically significant difference between sexes with males showing a higher prevalence of moderate to severe periodontitis than females (33.3%, 10.3%). (*p* = 0.008) (Table 6).

## 11. Vertical Bone Loss

**Figure 4:**
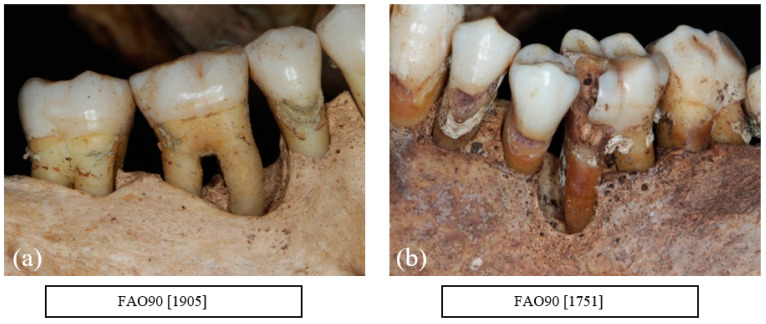
(**a**) FAO90[1905] Vertical bone loss mesial of a lower right mandibular first molar (LR6) with evidence of class III furcation involvement (courtesy of the Museum of London) and, (**b**) FAO90[1751] Vertical bone loss mesial of a lower left mandibular first molar (LL6) with a deep carious lesion and pulp exposure (evidenced by the defect around the exposed root) which could be associated with an endo-perio lesion (courtesy of the Museum of London).

A total of 4.3% of the teeth were affected by vertical bone loss. Vertical bone loss was usually localised around individual teeth or between two neighbouring teeth and generally affected both first and second molars with a few cases affecting the premolars and canines. One individual showed evidence of vertical bone loss in the incisor region. The size and the shape of the defect varied from a narrow interproximal defect to a large defect extending up to the apex (Figure 4a,b).

## 12. Other Dental Pathologies and Anomalies

**Figure 5:**
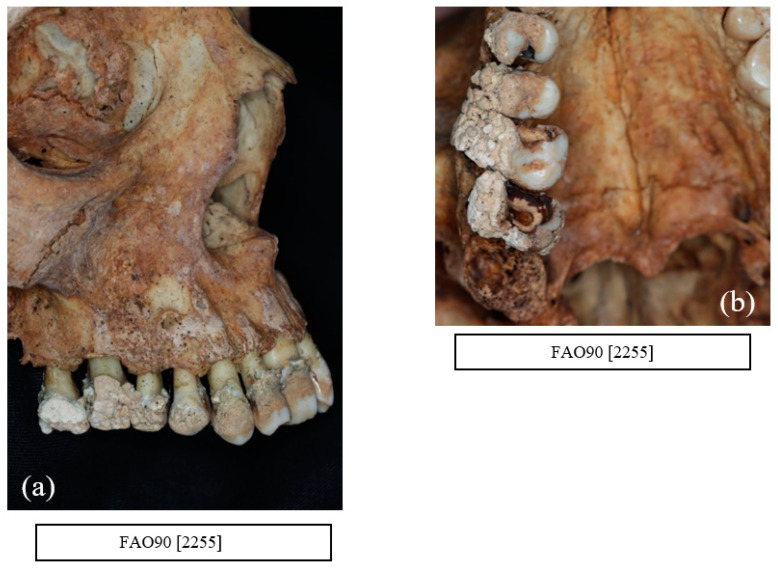
(**a**) FAO90 [2255] Female of age group 36–45 years with extensive deposit of supra gingival calculus covering the crowns of the upper maxillary teeth and (**b**) FAO90 [2255] Occlusal view of the same individual with evidence of occlusal calculus in the upper right quadrant. The right second molar (UR7) shows a gross carious lesion with pulp exposure (courtesy of the Museum of London).

**Figure 6:**
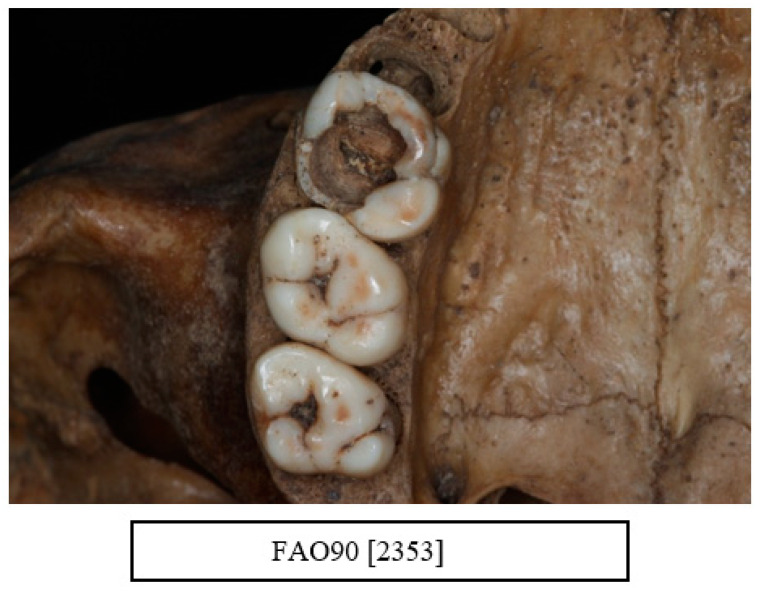
Occlusal view showing an extensive carious lesion in the upper right first molar (UR6) extending to the pulp chamber (courtesy of the Museum of London).

**Figure 7:**
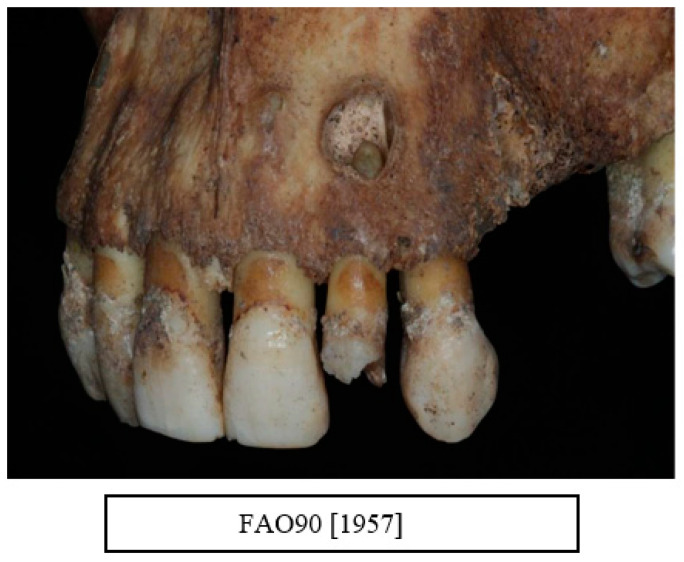
A periapical lesion associated with a retained root of an upper left second incisor tooth (UL2) (courtesy of the Museum of London).

**Figure 8:**
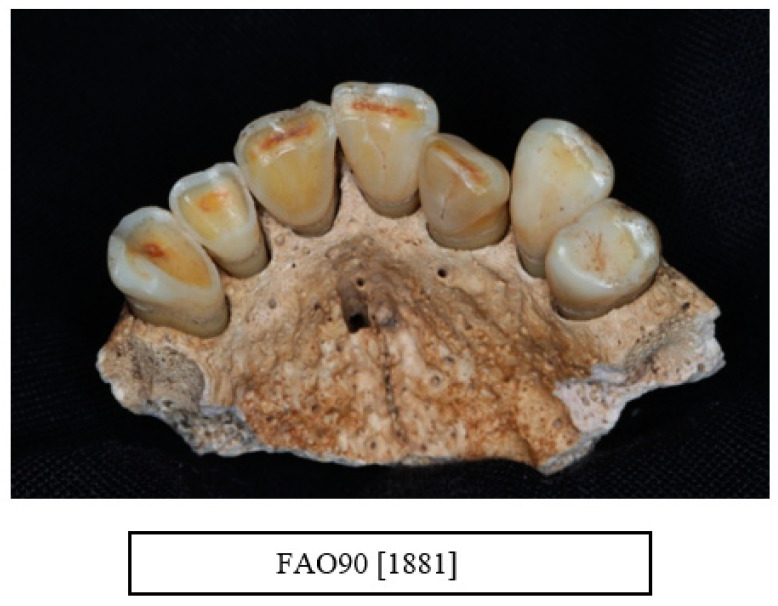
Tooth wear in the maxillary teeth of a male aged between 36–45 years. Note the different degree of wear pattern in the teeth. The anterior segment (UR3-UL2) shows extensive tooth wear with exposure of the dentine (code 3) and the upper left canine (UL3) has a flattened cusp (code 2) (Raitapuro-Murray et al. [3] index) (courtesy of the Museum of London).

**Figure 9:**
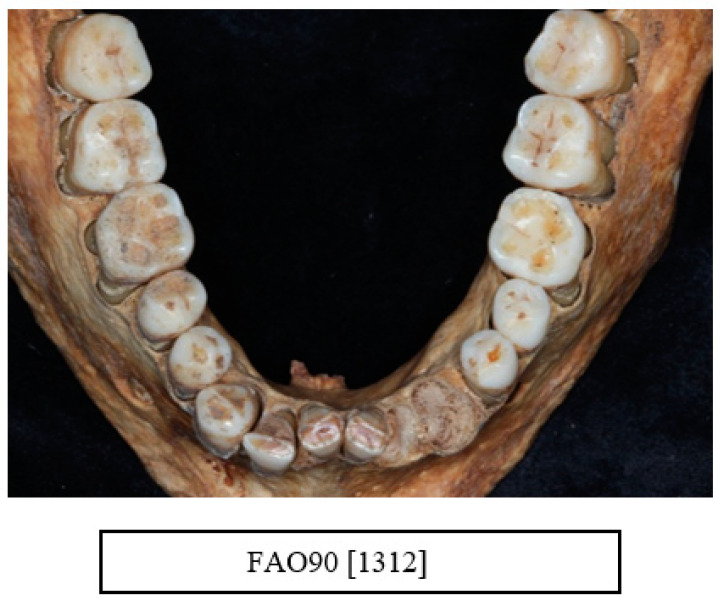
A 36–45-year-old male with generalised occlusal attrition in the mandible (lower jaw). Note the degree of tooth wear associated with the pattern of tooth eruption (courtesy of the Museum of London).

Other dental pathologies such as caries, calculus, and tooth wear are shown in Table 7 and Figure 4, Figure 5, Figure 6, Figure 7, Figure 8 and Figure 9.

Calculus was observed in all individuals in the sample. The amount of calculus deposits varied from discrete with small deposits in the region of CEJ to extensive calculus covering the entire tooth (Figure 5a,b).

Carious lesions were evident in all age groups. All types of carious lesions were observed in this sample including root caries, interproximal caries, and occlusal caries. A total of 89.5% of the individuals were affected with a carious lesion, with a mean number of 4.8 lesions. The most affected teeth were the first molars followed by the second molars (Figure 4b, Figure 5b, Figure 6 and Figure 7)

**Figure 10:**
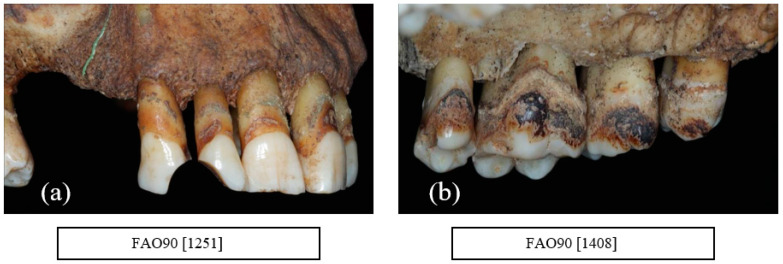
A total of 14% of individuals showed signs of smoking as indicated by (**a**) a pipe facet between the upper right second incisor (UR2) and canine (UR3) (FAO90 [1251]) and (**b**) staining of the maxillary teeth (FAO90 [1408]) (courtesy of the Museum of London).

A total of 13 males and 2 females (14%) were observed to exhibit signs of smoking including staining. Smoking was indicated by a pipe wear facet where a clay pipe left a wear facet that was circular in shape mainly affecting the upper and lower incisors and canine (Figure 10a,b).

## 13. Evidence of Recognised Dental Treatment

**Figure 11:**
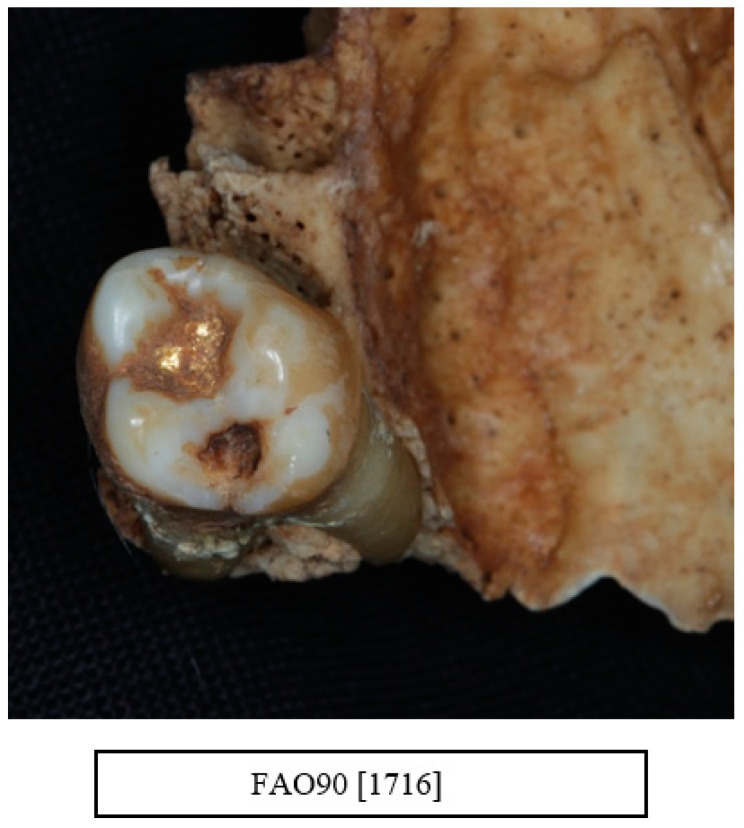
One individual presented with a gold restoration in a maxillary molar tooth, which appeared to be the only sign of any dental treatment other than extraction (courtesy of the Museum of London).

In the sample of skulls selected from the St Bride’s Lower Churchyard collection of 18–19th century skulls in London, the only evidence of any dental treatment other than extraction was one gold restoration (Figure 11).

## 14. Discussion

### 14.1. Overview

The St Bride’s Lower Churchyard is one of the largest recorded post-medieval samples in London. During this period, child mortality was high, and overall, 32% of the population did not survive into adulthood, with 85% of all children dead before the age of five years. Most of the observed pathologies in this sample were metabolic disorders such as rickets and scurvy. Trauma was very common, with rib fractures and nasal fractures as well as recognized infectious diseases included treponematosis, syphilis, and tuberculosis, although most infections were notated as non-specific infections [12,16]. Deceased adults in the St Bride’s Lower Churchyard cemetery were of a low socioeconomic status coming mainly from either a prison or workhouse population. This observation may lead to the assumption that these individuals were under stress and had poor nutrition, which in turn may have contributed to an increase of disease prevalence, including periodontitis [10].

### 14.2. Methodological Issues When Determining the Prevalence of Periodontal Disease in Dried Skulls

The present study demonstrated that there was a relatively high level of moderate to severe periodontitis in the St Bride’s Lower Churchyard 18–19th century sample together with significant and widespread oral pathology including caries and periapical lesions. It was evident when designing the present study, however, that the discrepancies between the various case definitions may impact on the actual prevalence values for periodontitis in the sample. Therefore, it was agreed to accept the methodology used by Raitapuro-Murray et al. [3], where a case definition was set to identify individuals with a level of periodontal disease which in the modern environment would be diagnosed as moderate to severe periodontitis as well as attempting to exclude false positives. Based on case definition I, 22 individuals were identified with moderate to severe periodontitis (21.0%). According to case definition II, 26 individuals were identified with moderate to severe periodontitis (24.8%). In both case definitions, older individuals showed a higher prevalence of the disease. The prevalence of individuals from the oldest age group affected by moderate to severe periodontitis was 41.9% and 27.9% respectively (Table 6). By way of comparison with the earlier Romano-British sample (200–400 AD) (5.6%) [3], the prevalence of moderate to severe periodontitis in the present study appeared higher, although this figure was considerably less than that observed in a modern population. Individuals from both the Romano-British and St Bride’s Lower Churchyard samples also presented with poor oral hygiene as was evidenced by the presence of calculus in all individuals in the two sample populations. One possible reason for the increase in the prevalence of periodontal disease between these two distinct population samples may be due to an increase in currently recognised risk factors such as smoking, stress, low socioeconomic status, and malnutrition [3]. The pattern of the disease observed between the two samples could also be influenced by their different genetic background [9].

### 14.3. The Impact of Ante-Mortem (AMTL) and Post-Mortem Damage (Including Anti-Mortem and Post-Mortem Tooth Loss) in Estimating the Level of Disease in Ancient Populations

Studying periodontal disease in an ancient population with dried skulls, however, can be challenging. For example, both ante-mortem (AMTL) and post-mortem damage (including anti-mortem and post-mortem tooth loss) was the most common problem faced in this study that led to an underestimation of the various dental pathologies identified in the skull collection including periodontal disease, caries, and calculus. For example, the skeletal remains from the original collection (*n* = 516) were excluded for the following reasons: (1) 228 missing skulls, (2) 43 adults were unclassified or under 18 years of age, (3) 84 adults were either edentulous or had post-mortem damage, and (4) 56 adult skulls were unexamined due to time constraints. Some of the teeth were present but could not be placed in the correct position by RA due to several factors such as extensive tooth wear, extensive caries, or a damaged tooth socket. In the present study the prevalence of AMTL was higher in older individuals with average of 8.8 teeth lost per individual. Both the maxilla and the mandible showed similar numbers of AMTL (average 3.5 and 2.8), the reason for tooth loss in this sample may have been due to trauma, caries, extensive tooth wear, periapical pathology such as an abscess, or periodontal disease. Individuals who were edentulous were excluded from this study. In retrospect, this decision may have resulted in the underestimation of the total AMTL in the population, especially in the older age group.

Although most of the skulls were in good condition, the alveolar margins were commonly damaged, during burial, excavation, and processing, which may have been a major cause of post-mortem tooth loss [13,17]. Single rooted teeth were mostly affected, which could be due to the root anatomy and loss of the soft tissue including the periodontal ligament [18]. A common problem identified in the present study was damage to the buccal bone including fractures, which led to the exclusion of the teeth and as such led to an underestimation of the prevalence of bone loss due to periodontal disease. Furcation involvement was also common in the St Bride’s sample, and periapical lesions (bone defect) were observed in half of the sample with different shapes and severity ranging from a small defect to a large defect involving more than one tooth, which was a similar observation in the Romano-British sample [3].

### 14.4. Attempts to Overcome the Problem of Inaccurate Linear Measurement of the Distance between the CEJ and AC When Examining Dried Skulls

One of the previous issues in assessing the prevalence of periodontal disease (periodontitis) in ancient populations was the accuracy of the linear measurement of the distance between the CEJ and AC in the skull collections. This measurement, however, ignored the continuous eruption of the teeth during the individual’s life and consequently, overestimated the prevalence of periodontitis [3]. To overcome this possibility, the Goncalves et al. [9] methodology was used in the present study for the CEJ-AC measurements. According to Goncalves et al. [9], subtracting 2 mm corresponded to the CEJ-AC distance which was considered as normal in healthy young individuals based on the radiographic imaging of the teeth [19]. For healthy individuals over 45 years several investigators have recommended that an average of 3 mm should be considered normal [20,21]. The rationale for these recommended measurements was that measurements over this average value would be considered as alveolar bone loss due to periodontal disease [9]. Except for the youngest age group, teeth with a higher level of tooth wear presented a similar mean level of CEJ-AC distance when compared to teeth with a low tooth wear grading. In the youngest age group, there was a large difference between the two samples (wear 0/1 and wear 2/3), which may have occurred due to the small sample size. Furthermore, one of the individuals in this age group had a large amount of ante-mortem tooth loss and extensive tooth wear, which was unusual for an individual of that age. According to the general pathology data, this individual also had trauma with an oblique non-union fracture of the right femur together with evidence of bone pathology which may have influenced the bone remodelling process. The results from the St Bride’s Lower Churchyard sample agreed with the study of a UK late-medieval population study [9] in that, regardless of the wear level, the CEJ-AC was always similar, and any measurements obtained were more likely to be bone loss due to persistent inflammation because of the periodontal disease progression. It should however be acknowledged that the index used in the Goncalves et al. [9] study was the same as that used in the Smith and Knight study [22], whereas in the present study the index used was the same as that employed in the Raitapuro-Murray et al. [3] study. Larsen [23] also indicated that, in general, there appeared to be an increase in periodontal disease (over time) with a transition from traditional to western-style processed diets. For example, past populations consuming large amounts of plant carbohydrates or processed foods have higher rates of periodontitis compared to foragers with substantial amounts of animal protein in their diets.

### 14.5. Problems When Comparing Disease Prevalence in Ancient and Modern Populations

Variation in the methodology, case definition, and the statistical interpretation of the data from previous studies made comparison of the results difficult, particularly regarding the use of a case definition to assess periodontitis. In the present study the methodology of Raitapuro-Murray et al. [3] was used, and for example, in case definition I there were no individuals affected in the 36–45 years age group, whereas nine individuals were considered as affected using case definition II. The reason for the difference was that for case definition I, four quadrants were required to be considered as periodontally affected and in this age group several of the quadrants were edentulous. The other reason was that in some individuals only one jaw was present, therefore, the individual was excluded from the affected category. It should be noted, however, that the measurements recorded in the present study represented an estimation of bone loss and therefore cannot be viewed as an accurate representation of periodontal disease progression.

Comparison of the prevalence rates from the various studies proved problematic due in part to differences in methodology and case definitions when recording the prevalence of periodontal disease from dried skulls (with the absence of soft tissue including periodontal pockets) in a historical collection of human remains compared to living individuals within a modern-day population. For example, as previously mentioned, the prevalence of moderate to severe periodontitis in the 18–19th century skull sample was 21–24%, which was higher than in the Romano-British sample (5.6%) [3], whereas the prevalence of periodontitis in adults over 30 years (as classified by the Centers for Disease Control and Prevention (CDC)/American Academy of Periodontology (AAP) case definitions) was 7.8% for severe periodontitis and 34.4% for non-severe (mild to moderate) periodontitis respectively [24]. Similar prevalence rates were also recorded in the Adult Dental Health Survey in the United Kingdom (excluding Scotland) with 9% of dental adults with severe periodontitis and 37% with pocketing of 4–6 mm respectively [25].

In the present study, there was a low number of teeth presenting with vertical bone loss. Cases of localized vertical bone loss of pulpal origin were extremely severe and extensive and were usually associated with deep carious lesions and pulpal exposure due to caries, extensive tooth wear, or tooth fracture. Other reasons for localized vertical bone loss may include food or foreign object impaction, trauma, or root fracture [3]. Most of the St Bride’s sample suffered from attrition with tooth wear ranging from mild to extensive wear. There were no differences between the age groups regarding the severity of tooth wear. In several individuals the tooth wear was so severe that it led to a pulpal exposure, which was also observed in the Romano-British sample [3]. The index used for recording tooth wear in the present study however was not sensitive in detecting any differences between the age groups since most of the population, even those in the younger age group, had exposed dentine which in some cases exposed the pulp chamber. Attrition is the normal result of tooth use, either for mastication or as a tool. The primary cause of tooth wear has been previously assumed to be dietary in nature with the fibrous or abrasive nature of food [3,26] although a secondary cause of tooth wear, historically, was the use of teeth as a tool.

Evidence of heavy smoking was observed in 14% of the sample. Clay pipes were used in the 18–19th century which left a clear circular wear pattern on the tooth surface. According to Albandar et al. [27], pipe smoking may have similar adverse effects on periodontal health and tooth loss as cigarette smoking. Furthermore, smoking increased both the prevalence and severity of periodontal disease in susceptible individuals and exposure was associated with a 2- to 3-fold increase in the odds ratio of developing periodontitis [28].

A high percentage of skulls in the present study also suffered from significant and widespread oral pathology including dental caries (89.5%; 4.8 mean per tooth) and periapical lesions (40%; 0.7 mean per tooth affected) (Table 7). Dental caries varied between the skulls and the most affected teeth were the first molars followed by the second molars (involving mainly the occlusal and root surfaces). The St Bride’s sample was previously studied for the prevalence of caries by Mant and Roberts [29]. The prevalence of dental caries in the total population was 78.9%, and of those skulls identified with dental caries, 26.5% of all teeth present were affected. These investigators compared the data to other populations and concluded that there were no significant differences in the rate(s) of caries in individuals either by gender or social status [29]. More recently, Smith [30] compared post-medieval and modern-day caries exposure of adults living in the East London borough of Tower Hamlets, UK, using recorded data from human remains excavated from New Churchyard located under a major London railway station (Liverpool Street) and data from the East London Oral Health Inequality study (ELOHI) [31]. From the results of this comparative data, it was evident that there were significantly lower rates of dental caries (decay) in a post-medieval London sample than in a modern-day population. One of the problems of comparing data from historical skull collections and modern populations is the method of data collection, which has changed. For example, in the St Bride’s [29], Raitapuro-Murray et al. [3], and the New Churchyard [30] studies caries was not collected for analysis using the Decayed, Missing, Filled Teeth index (DMFT), whereas in the modern population (ELOHI) study (cited by Smith [30,31]), examiners were trained and calibrated in the use of the index resulting in a good level of examiner reproducibility. It should be noted, however, as Smith [30] indicated in his paper, that he was unable to compare the raw data from the ELOHI study when comparing the New Churchyard study and a representative modern-day sample (ELOHI). Nevertheless, the recorded caries rate in the Romano-British sample (43.9% to 75.4%; mean teeth affected 1.5–2.3 depending on the age cohort) and post-medieval samples (St Bride’s 89.5% (*n* = 94), 4.8 mean per tooth and New Churchyard 27.9%, F: average 4.5 decayed teeth and 6 missing teeth, M: average 4.3 decayed teeth and three missing teeth) was lower than that of a more modern comparable sample (ELOHI (“White British” M/F) DMFT mean (95% CI): 13.47 (12.52–14.42)) despite the differences in data collection [3,31,32].

The progression from a relatively low caries rate as shown in the Romano-British [3] and the post-medieval samples (St Bride’s and New Churchyard) culminating in a higher caries rate as evidenced in a more modern population [31] can be associated with poor oral hygiene and a cariogenic diet. For example, the use of sugar throughout the 18th century was a key factor in the development of caries, and its increase in use is directly linked to an increase in tea drinking throughout the UK, resulting in a carbohydrate and soft diet that allowed plaque accumulation around the teeth. This trend has continued in a modern-day population with access to a more cariogenic diet [30]. It should also be acknowledged that the frequency of the caries rate in the present study could be underestimated due to both ante-mortem and post-mortem tooth loss as well as the short life spans of the individuals within the sample. Furthermore, it is evident from recent studies that the frequency and distribution of caries and ante-mortem tooth loss increased in ancient populations with a longer life span [17,29,30,31,33]. In the present study there was no evidence of ‘routine’ dental treatment other than extraction, which may be indicative of the low socio-economic status of the individuals in the sample, an observation that appears to be supported by Hillam [34] who reported that there was limited data of any conservative dental treatment practiced in both Britain and mainland Europe up to the end of the 18th century.

Although the study of ancient populations may contribute to an understanding of both the epidemiology and natural history of a disease there is a need for investigators to agree on guidelines using the same methodologies (including training and calibration) and case definitions when examining periodontal disease and other oral pathologies in dried skull collections.

## 15. Conclusions

The study of 105 (66 M;39 F) deceased adults over 18 years of age demonstrated that there was a relatively high level of moderate to severe periodontitis in the St Bride’s Lower Churchyard sample together with significant and widespread oral pathology including caries and periapical lesions. The prevalence of the periodontal disease was higher than that reported in a Romano-British sample but of a similar prevalence as observed in modern populations. The observed increase in the prevalence of periodontal disease between these two distinct samples may be due to factors such as poor oral hygiene and an increase in currently recognised risk factors such as smoking, stress, low socioeconomic status, and malnutrition.

## Figures and Tables

**Table 1:** Data collected for the study.

Data Collected from Each Skull
Age
Sex
Bone present (mandible/maxilla)
Teeth present
Teeth lost ante-mortem (AMTL)
Teeth lost post-mortem
Tooth surface loss
Bone loss
Pattern of bone loss (horizontal or vertical)
Bone defect
Calculus
Caries
Other pathologies/anomalies (furcation, enamel hypoplasia, enamel projection)

**Table 2:** Dental pathology/anomalies (presence/absence).

Dental Pathology/Anomalies	Recorded as
Caries	Present or absent per tooth
Calculus	Present or absent per tooth
Furcation of the tooth	Present or absent per tooth
Periapical lesion	Present or absent per tooth
Pulp exposure	Present or absent per tooth
Fracture	Present or absent per tooth
Dehiscence	Present or absent per tooth
Fenestration	Present or absent per tooth
Enamel hypoplasia	Present or absent per skull
Supernumerary teeth	Present or absent per tooth
Enamel projection	Present or absent per tooth

**Table 3:** Ante-mortem tooth loss (mean).

Age Group	Number of Individuals	% of Individuals	Number of Teeth
18–25 years	5	83.3%	4.8
26–35 years	19	95.0%	4.1
36–45 years	31	86.1%	3.9
46+ years	40	93.0%	8.8
Total	95	90.5%	6.0

**Table 4:** Post-mortem tooth loss (mean).

Age Group	Number of Individuals	% of Individuals	Average Number of Teeth
18–25 years	5	83.3%	6.8
26–35 years	18	90.0%	5.5
36–45 years	28	77.8%	5.0
46+ years	34	79.1%	3.6
Total	85	81.0%	4.6

**Table 5:** Horizontal bone loss by age group.

Age Group	Number of Adults	% Adults with Bone Loss
18–26 years	2	33.3
26–35 years	7	35.0
36–45 years	13	36.1
46+ years	17	39.5
Total	39	37.1

**Table 6:** Cases with moderate to severe periodontitis (age and sex) based on case definition I and case definition II.

**Age Group**	**No. of Affected Adults (Case Definition I)**	**% Affected** **Adults (Case Definition I)**	**No. of Affected Adults (Case Definition II)**	**% Affected Adults (Case Definition II)**
18–25 years	2	33.3	2	33.3
26–35 years	2	10.0	3	15.0
36–45 years	0	0.0	9	25.0
46+ years	18	41.9	12	27.9
Total	22	21.0	26	24.8
Gender				
**Sex**	**No. of Affected Adults (Case Definition I)**	**% Adults (Case Definition I)**	**No. of Affected Adults (Case Definition II)**	**% Adults (Case Definition II)**
Male	15	22.7	22	33.3
Female	7	17.9	4	10.3
Total	22	21.0	26	24.8

**Table 7:** Dental pathologies and anomalies.

Dental Pathologies and Anomalies	Number of Adults	% of Adults	Mean Teeth Affected
Tooth wear (attrition)	105	100	16.7
Calculus	105	100	19
Furcation of the tooth	92	87.6	3.6
Caries	94	89.5	4.8
Periapical lesion/bone loss	42	40	0.7
Pulp exposure	53	50.5	1
Enamel projection	17	16.2	0.3
Dehiscence	22	21	0.4
Fenestration	55	52.4	1.3

## Data Availability

The skeletal collection is archived and the data available from the Museums data base.
